# c.7156C > T p.(Gln2386*) variant causes loss-of-function of the *USP9X* gene in a female-restricted X-linked syndromic intellectual disability: a case report

**DOI:** 10.1186/s13256-025-05456-z

**Published:** 2025-08-01

**Authors:** Talyta Alves da Silva Campos, Alex Honda Bernardes, Irene Plaza Pinto, Hiane Aparecida da Silva Teixeira, Juliana Ferreira da Silva, Victor Cortázio do Prado Santos, Raffael Zatarin, Aparecido Divino da Cruz

**Affiliations:** 1 Replicon Research Center, Master Program in Genetics, School of Medical Science and Health, Pontifical Catholic University of Goiás, Goiânia, Goiás Brazil; 2 Clinical Genetics Service, Center for Rehabilitation and Readaptation Dr. Henrique Santillo, State Health Secretary of Goiás, Goiânia, GO Brazil; 3 Graduate Program in Genetics and Molecular Biology, Federal University of Goiás, Goiânia, GO Brazil; 4 Medical School, Federal University of Goiás, Goiânia, Goiás Brazil

**Keywords:** MRXS99F, *USP9X*, Neurodevelopmental disorder, Developmental delay, Case report

## Abstract

**Background:**

Female-restricted X-linked syndromic intellectual developmental disorder-99 is an ultrarare neurodevelopmental disorder linked to X, manifesting in female individuals due to mutations in the *USP9X* gene. It is characterized by developmental delays, behavioral alterations, and moderate-to-severe intellectual disability. The *USP9X* gene plays critical roles in protein turnover and the regulation of essential pathways during neural development. This work describes the case of a Brazilian patient with female-restricted X-linked syndromic intellectual developmental disorder-99 with a variant not found in databases such as Decipher and ClinVar. Information was obtained from the Center for Rehabilitation and Readaptation Dr. Henrique Santillo electronic medical record system, and exams were conducted by partner laboratories of the Unified Health System. Documenting cases in different populations enriches the knowledge of genetic variations, guides personalized treatments, and expands the field of medical genetics, underscoring the importance of this study.

**Case presentation:**

A 3-year-old female patient of Pardo admixed ethnicity from northern Brazil was referred to the Center for Rehabilitation and Readaptation Dr. Henrique Santillo for suspected genetic disorders. The child was born after an uneventful pregnancy but faced neonatal complications, including cardiopulmonary arrest and jaundice, requiring intensive care unit admission. She was diagnosed with nonprogressive encephalopathy and neuropsychomotor developmental delay. Additional tests revealed structural anomalies, such as corpus callosum agenesis and congenital hip dysplasia. Various genetic tests were performed, but only whole exome sequencing revealed a pathogenic variant in the *USP9X* gene, associated with female-restricted X-linked syndromic intellectual developmental disorder-99.

**Conclusion:**

We report the case of a child with a heterozygous pathogenic variant in the *USP9X* gene, located at Xp11.4 and presenting a wide range of phenotypes. The cytosine-to-thymine substitution resulted in a premature stop codon, causing female-restricted X-linked syndromic intellectual developmental disorder-99. The mutation leads to protein function loss due to haploinsufficiency, resulting in a dominant X-linked disorder. Loss-of-function mutations in the *USP9X* gene cause intellectual disability and congenital anomalies, with several craniofacial anomalies observed in the patient. Despite the *de novo* nature of most loss-of-function variants, maternal testing is crucial for estimating recurrence risk. Genetic investigation confirmed the variant’s pathogenicity, highlighting diagnostic challenges and the importance of genetic research in understanding and managing female-restricted X-linked syndromic intellectual developmental disorder-99.

**Graphical Abstract:**

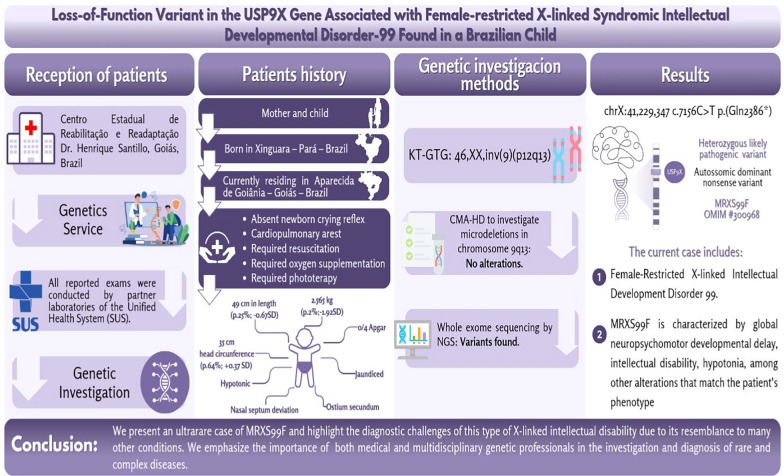

## Background

Intellectual disability (ID) is characterized by significant limitations in both intellectual functioning and adaptive behavior, manifesting before the age of 18 years. The clinical identification of ID involves neuropsychological evaluation, with an intelligence quotient (IQ) measurement below 70. ID is classified in four levels, namely mild, moderate, severe, and profound, on the basis of the severity of limitations and the level of support required for the individual’s adequate adaptation to their environment [1, 2].

ID has an approximate global prevalence of 2.4% among live births, being 54% more frequent in male individuals. The etiology of ID is multifactorial, encompassing genetic and environmental factors. Genetic factors include alterations in crucial genes for neurological pathways, while environmental factors include, for instance, prenatal malnutrition and exposure to teratogenic agents [3].

Among the various patterns of genetic inheritance associated with ID, X-linked inheritance is particularly intriguing due to its peculiarities between male and female individuals. In general terms, most families exhibit a clear X-linked segregation pattern, where male individuals are affected, and female individuals are unaffected carriers or mildly affected. In contrast, the number of identified X-linked genes, in which *de novo* mutations specifically cause ID in female individuals, is limited [4, 5]. In this context, clinical evaluations are essential for a comprehensive understanding of the pathogenesis of X-linked inheritances [6].

Female-restricted X-linked syndromic intellectual developmental disorder-99 (MRXS99F), OMIM #300968 [7], is an ultrarare neurodevelopmental disorder, with an estimated incidence of 1:1,000,000 live births, which manifest as a dominant X-linked trait [8]. Phenotypically, MRXS99F is associated with developmental delay, behavioral alterations, and moderate-to-severe intellectual disability. Genotypically, the condition is caused by heterozygous variations in the *USP9X* gene (Ubiquitin Specific Peptidase 9, X-linked – OMIM *300072), located in the Xp11.4 region [7].

The *USP9X* gene encodes a highly conserved deubiquitinating enzyme that processes ubiquitin precursors and ubiquitinated proteins, playing crucial roles in protein turnover and the regulation of transforming growth factor-beta (TGF-beta) and bone morphogenetic protein during fetal and neural development [2]. Alterations in *USP9X* gene in female individuals are associated with neurodevelopmental disorders, hypoplastic corpus callosum, congenital malformations, and dysmorphic facial features. Affected female individuals typically present with heterozygous loss-of-function variants, both inherited and *de novo*, while affected male individuals usually carry missense variants, which can be either *de novo* or inherited from an unaffected mother. Loss-of-function variants in male individuals have never been reported, as it is believed that total loss of protein function is incompatible with life [9, 10].

The current case report presents a Brazilian female patient diagnosed with MRXS99F with exclusive phenotypes and a variant not found in the databases; this is an ultrarare case with clinical and genetic relevance, as it details the genetic variant it contributes to the understanding of the molecular biology of the condition and expands the field of medical genetics. Documenting cases in different populations enriches the knowledge regarding genetic variation in different contexts and populations.

This article can serve as a starting point for future investigations into the *USP9X* gene and its implications, encouraging further studies and discoveries. Additionally, it provides details that can help clinicians and geneticists to identify the condition more accurately in other patients. The information about the genetic variant can guide the development of personalized therapies and specific treatments, equipping doctors and healthcare professionals with detailed knowledge to improve the management and care of affected patients.

## Methods

The pieces of information presented herein were acquired from the electronic medical record system of a patient treated at the Center for Rehabilitation and Readaptation Dr. Henrique Santillo (CRER), in Goiás, Brazil, a public tertiary care hospital. All reported exams were conducted by partner laboratories of the Brazilian Unified Health System (*Sistema Único de Saúde*, SUS). The research in question has been submitted and approved by the ethics committee of the Pontifical Catholic University of Goiás (PUC-GO) under approval number 6.057.949, following the ethical principles of the Declaration of Helsinki.

## Case presentation

A 3-year-old female patient of Pardo admixed ethnicity, assisted by the medical and multidisciplinary team at CRER, was referred to the care of medical genetics and genetic counseling service for the investigation of a possible genetic disorder. The child was originally from a northern state of Brazil, and at the time of this report, resided in Central Brazil.

The mother, G1P1A0, denied consanguinity and reported an uneventful pregnancy. Prenatal care started in the first trimester and fetal movement was felt at 20 weeks of gestation. An uneventful vaginal delivery at term, with 41 weeks and 1 day of gestation, followed uncomplicated stages of labor, birth, and delivering the placenta. The child was born weighing 2.565 g (p.2%; −1.92 SD), classified as small for gestational age, measuring 49 cm (p.25%; −0.67 SD), which was appropriate for gestational age, with a head circumference of 35 cm (p.64%; +0.37 SD), classified as normocephalic. Her Apgar score was 0/4, indicating severe distress requiring immediate resuscitation intervention three times on the day of birth due to cardiopulmonary arrest (CPA). She had persistent absence of crying reflex, was hypotonic, had difficulty sucking and rejection of breastfeeding, and required immediate medical care. The newborn had jaundice, underwent phototherapy for 5 days, and required oxygen therapy. The baby stayed in the intensive care unit (ICU) for 28 days. Neonatal screening tests were normal but for the immunological tests for infectious diseases. Positive results we observed for both rubella and toxoplasmosis immunoglobulin (Ig)G. During the neonatal period, a GTG-banding karyotype (KT-GTG) was performed, revealing 46,XX,inv(9)(p12q13)—a pericentric inversion on chromosome 9, considered a normal variation in the general population (Table 1). While at the ICU, an echocardiogram revealed an ostium secundum atrial septal defect (ASD), perimembranous ventricular septal defect (VSD), and moderate pulmonary hypertension.

**Table 1:** Presentation of tests, and their respective results, carried out on proband and parents

List of tests and their results		
Proband	Proband’s mother	Proband’s father
Neonatal screening tests—Normal	–	–
Neonatal rubella and toxoplasmosis IgG—Positive	–	–
Neonatal GTG-banding karyotype—Revealing 46, XX, inv (9) (p12q13)	GTG-banding karyotype—No abnormalities	GTG-banding karyotype—Revealing 46, XY, inv (9) (p12q13)
Neonatal echocardiogram—Revealed an ostium secundum atrial septal defect and perimembranous ventricular septal defect	–	–
1 year old—Cytomegalovirus/syphilis/human immunodeficiency virus (HIV) 1 and 2—All tests returned non-reactive for both IgG and IgM	Serologies for rubella and toxoplasmosis IgG—Positive	–
Computed tomography—Revealed agenesis of the corpus callosum, signs of bilateral incomplete hippocampal inversion, volumetric reduction of the cerebral white matter predominantly in the peritrigonal region, and enlargement of the foramen of Magendie associated with ectasia of the fourth ventricle and the cisterna magna	–	–
Spinal X-ray—Revealing pronounced dorsolumbar scoliosis with dorsal convexity to the right and lumbar to the left, with the dorsal curvature being the primary one	–	–
Pelvic X-ray—Showing flattening and verticalization of the right acetabular surface and superolateral dislocation of the right femoral head, findings consistent with congenital hip dysplasia associated with right-sided dislocation	–	–
Ultrasonography of the urinary system—Showing kidneys with normal dimensions but dilation of their respective pelves and proximal ureters	–	–
High-resolution chromosomal microarray analysis—No alterations	–	–
Whole exome sequencing—Revealed a variant in the *USP9X* gene at position: chrX: 41,229,347, variant: c.7156C > T, consequence: p. (Gln2386*) ENST00000378308, in heterozygosity, classified as probably pathogenic	–	–

A hyphen (–) has been added to replace exams that have not been carried out.

At 28 days old, the patient underwent septoplasty, performed by an otolaryngologist surgeon, and remained hospitalized for an additional 17 days to improve her condition and achieve independence from oxygen therapy. At 6 months old, an ulcer was observed in her left eye, leading to two corneal transplants and temporary bilateral tarsorrhaphy, and decreased eye blinking and lubrication. The first transplant occurred at 9 months and the second at 15 months.

At 8 months, following a consultation with a neuropediatrician due to neuropsychomotor developmental delay (NPMDD), she was diagnosed with nonprogressive encephalopathy. The child exhibited delay in all her pediatric developmental milestones and some were never acquired, such as speech and walking, and she is totally dependent for her activities of daily living.

In 2019, at less than 1 year old, further tests were conducted for infectious diseases, including rubella, toxoplasmosis, cytomegalovirus, syphilis, and HIV 1 and 2. All tests returned nonreactive for both IgG and IgM. A cranial computed tomography (CT) scan revealed agenesis of the corpus callosum, signs of bilateral incomplete hippocampal inversion, volumetric reduction of the cerebral white matter, predominantly in the peritrigonal region, and enlargement of the foramen of Magendie associated with ectasia of the fourth ventricle and the cisterna magna (Table 1).

**Fig. 1:**
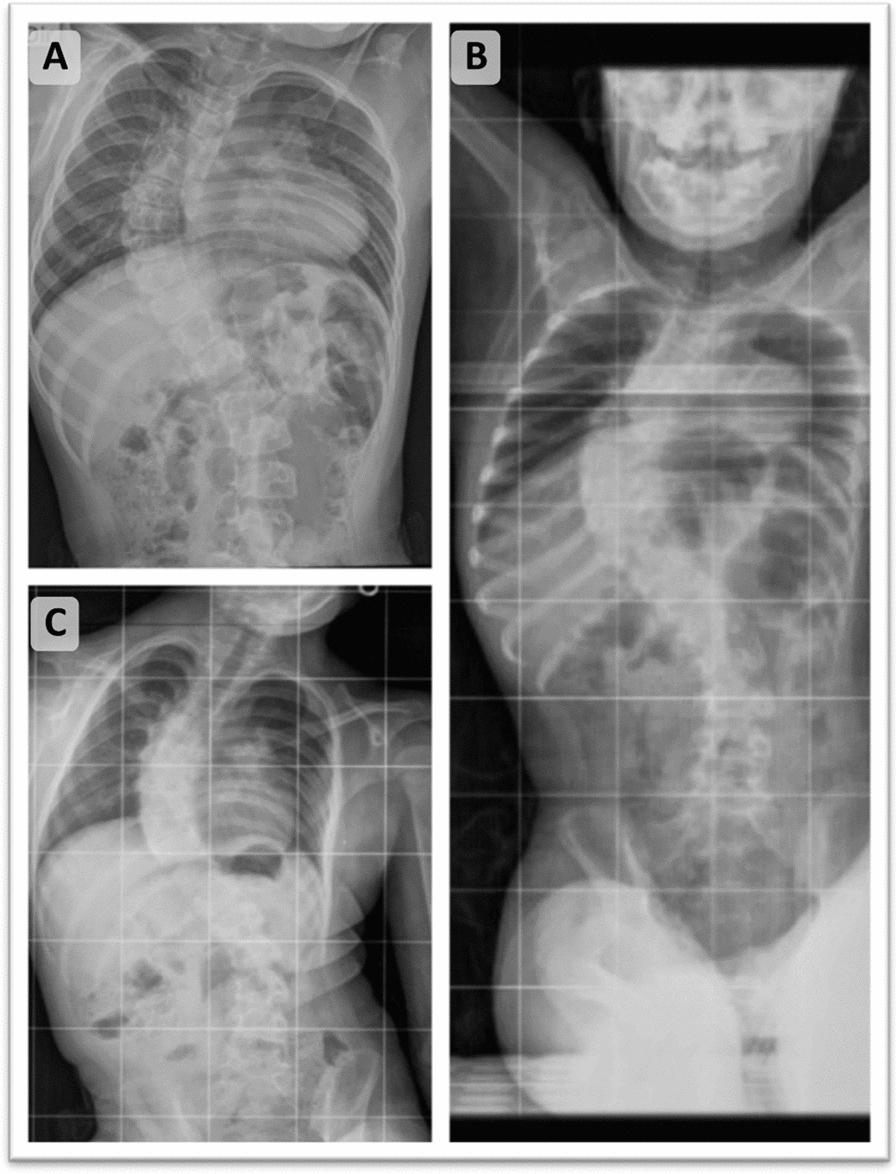
**A**–C* Spinal X-ray: pronounced dorsolumbar scoliosis with dorsal convexity to the right and lumbar to the left, with the dorsal curvature being the primary one associated with associated with female-restricted X-linked intellectual development disorder 99. *All panels are from the same examination

**Fig. 2:**
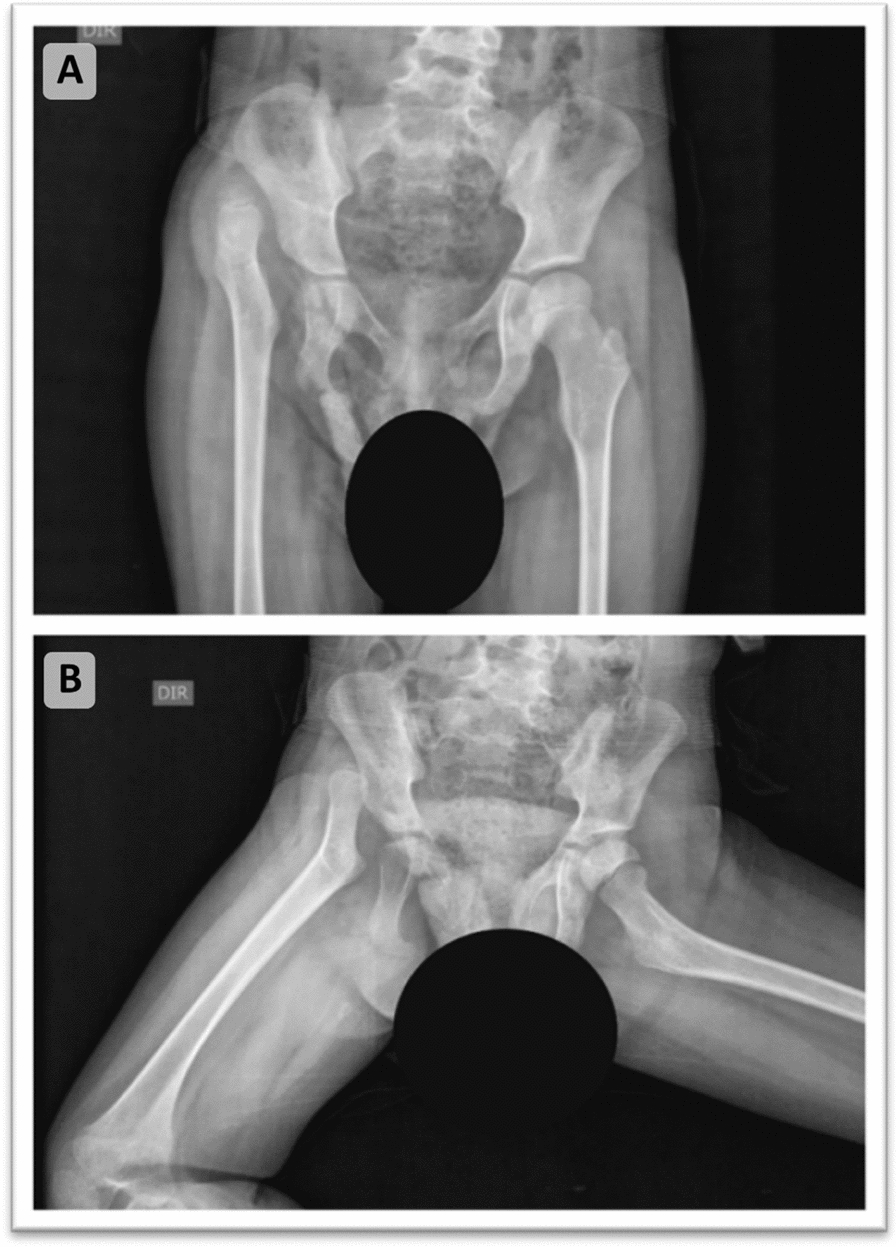
**A**, B* Pelvic X-ray: flattening and verticalization of the right acetabular surface, superolateral dislocation of the right femoral head, findings consistent with congenital hip dysplasia associated with right-sided dislocation in a girl with X-linked intellectual development disorder 99. *Both panels are from the same examination

Between the ages of 2 and 3 years, the following examinations were performed: spinal X-ray (XR), revealing pronounced dorsolumbar scoliosis with dorsal convexity to the right and lumbar to the left, with the dorsal curvature being the primary one (Fig. 1); pelvic XR, showing flattening and verticalization of the right acetabular surface and superolateral dislocation of the right femoral head, findings consistent with congenital hip dysplasia associated with right-sided dislocation (Fig. 2); and ultrasonography (USG) of the urinary system, showing kidneys with normal dimensions but dilation of their respective pelves and proximal ureters (Table 1). At the age of 4 years, she underwent orthopedic surgery to correct nonreducible cavovarus equinovarus feet.

Physical examination of the proband, conducted by the medical genetics staff at about 3.5 years old, revealed weight of 13.6 kg (p.15%; −1.04 SD), height of 94.2 cm (p.12%; −1.16 SD), and head circumference of 44.5 cm (p.1%; −5.26 SD). Both nonconsanguineous parents, who are healthy, denied similar cases, genetic disorders, intellectual disability, mental illness, stillbirths, neonatal deaths, or malformations in their families.

Initially, the clinical hypothesis included congenital rubella and/or toxoplasmosis syndrome. However, at the genetic counseling (GC) sessions the family was uneasy about the previous karyotyping results. Thus, follow-up with high-resolution chromosomal microarray analysis (CMA-HD) was proposed to investigate potential encrypted microdeletions in chromosome 9q13, which could help explain her phenotype. Additionally, further tests were requested, including KT-GTG for both parents and maternal IgG serology for rubella and toxoplasmosis, with test results presented in Table 1. Subsequently, the genetic counselor suggested whole exome sequencing (WES) by next-generation sequencing (NGS) as a potential test to assist the clinical team with the genetic diagnosis of the condition. The parents agreed to the test.

Conducted in 2024, at 5 years of age, WES revealed a variant in the *USP9X* gene at position: chrX:41,229,347, variant: c.7156C > T, consequence: p.(Gln2386*) ENST00000378308, in heterozygosity, classified as probably pathogenic and associated with X-linked intellectual development disorder 99, “female-restricted” (MRXS99F—OMIM #300968) [7]. MRXS99F is characterized by global neuropsychomotor developmental delay, intellectual disability, and hypotonia, among other alterations that match the patient’s phenotype. The test results were presented to the parents, and all doubts were clarified.

**Fig. 3:**
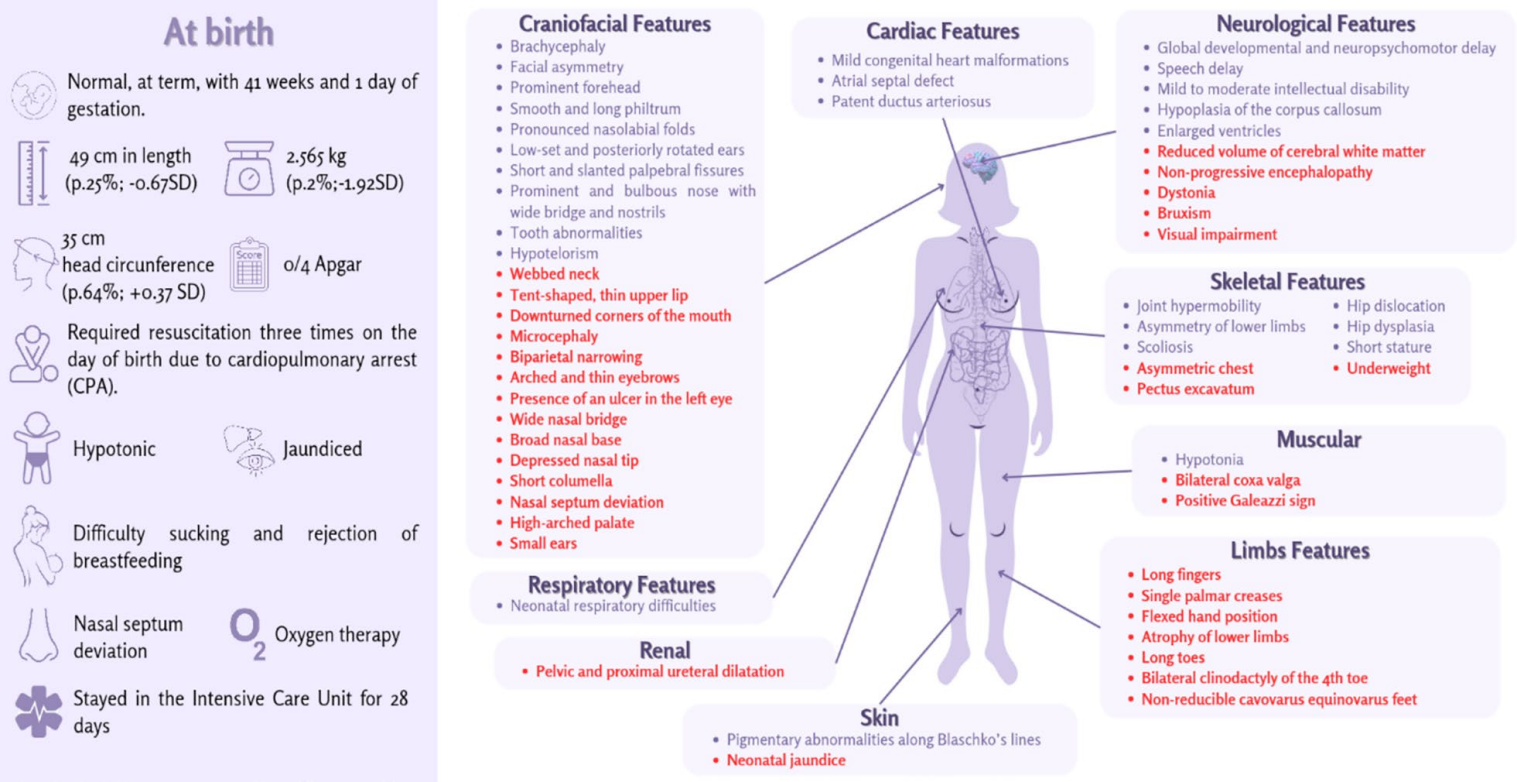
Summary of information on the patient’s birth and list of phenotypes identified. In red there are the exclusive phenotypes, which do not appear in the Human Phenotype Ontology and OMIM databases

In her latest session, conducted in 2024 at about 6 years of age, patient’s anthropometric measures were: weight of 16 kg (p.2%; −1.96 SD), height of 101 cm (p.1%; −3.04 SD), and head circumference of 46 cm (p.1%; −5.18 SD). All the proband’s phenotypes that were observed during the consultations can be seen in Fig. 3.

The case management was carried out through follow-up with a multidisciplinary team to assist the patient and her family. The specialties involved included medical geneticist, genetic counselor, orthopedist, physiotherapist, occupational therapist, speech therapist, dentist, otolaryngologist, ophthalmologist, neurologist, physiatrist, cardiologist, pediatrician, and nutritionist.

## Discussion

We report the case of a child with a heterozygous disease-causing variant in the *USP9X* gene, located on Xp11.4. The variation in the mRNA consisted of a substitution of cytosine to thymine at the codon 7.156. Consequently, glutamine was replaced by a premature stop codon truncating the protein at the amino acid residue 2.386. The transition was classified as probably pathogenic and associated with female-restricted MRXS99F. The nonsense variant caused loss of protein function due to premature termination of RNA in a locus susceptible to haploinsufficiency, resulting in a dominant X-linked disorder in the child.

There are, currently, 990 variants in the *USP9X* gene cited In ClinVar [11]. Of those variants, 244 (24.6%) were classified as pathogenic and probably pathogenic. Of the total, 30 (3%) were nonsense variants, also classified as pathogenic or probably pathogenic variants. At the time of preparing this report, the variant c.7156C > T has not yet been deposited in ClinVar [11].

In DECIPHER [12], we found 129 patients with variants in the *USP9X* gene, 31 (24%) sequence variants, 71 (55%) copy-number variants, 26 (20.2%) chromosomal anomalies, and 1 (0.8%) uniparental disomy. Of the 31 sequence variants, 15 were present in female patients and only 3 variants were an amino acid substitution with a premature stop codon, as seen in our patient. No nonsense variants were found in male patients in DECIPHER [12].

Studies indicated that loss-of-function (LOF) mutations in the germline of the *USP9X* gene cause intellectual disability (ID) and other congenital anomalies. Heterozygous LOF variants in female individuals lead to reduced *USP9X* mRNA expression, protein levels, and derived cells, contributing to various characteristic craniofacial abnormalities such as the ones observed in the reported patient [13, 14].

Figure 3 shows all the patient’s phenotypes, some of which have already been mentioned in the Human Phenotype Ontology (HPO) and OMIM databases, but most of which are phenotypes unique to our patient and are highlighted in red in the figure [7, 15].

Reijnders *et al*. [16] described the phenotypes of 17 patients carrying *de novo* LOF variants in *USP9X*. Some phenotypes observed in our patient were cited in the majority of the study’s patients, such as: intellectual disability or developmental delay (100% of cases), enlarged ventricles (73%), dental abnormalities (71%), scoliosis (65%), pigmentary abnormalities along Blaschko’s lines (65%), hypoplastic corpus callosum (62%), ocular abnormalities (59%), short stature (53%), hip dysplasia (47%), hypotonia (47%), and leg length discrepancy (41%) [16].

Most LOF variants in female individuals is *de novo*. However, Li *et al*. [14] reported the case of two non-twin sisters who inherited the variant from their mother, an asymptomatic carrier with reduced penetrance. This highlights that, despite the tendency for LOF variants in female individuals to be *de novo* with complete penetrance in 95% of cases, a note of caution is intended to genetic counselors and medical geneticists, who should confirm the variant origin by maternal testing, especially when disease-causing LOF variants are found in *USP9X*. Confirmation of the variant status should be done before estimating the recurrence risk of the disease [14]. In the current case, after pretesting genetic counseling, the mother of the proband declined testing as she was asymptomatic and had no reproductive interest. Thus, for the child reported here, *de novo* origin with unconfirmed parentage was assumed.

Approximately 15% of genes related to intellectual disability are located on the X chromosome, which comprises about 5% of the human genome. In female individuals, the presence of a second X chromosome leads to the inactivation of one of them, and in cases of mutations, this inactivation preferentially occurs in the mutant X chromosome in most cells [17]. However, Vianna *et al*. [17] estimate that 12–23% of X-linked genes escape inactivation, being expressed bilaterally. This is the case with the *USP9X* gene, which escapes inactivation and is expressed bilaterally, although heterozygous LOF variants in this gene are not compensated by the activity of the non-mutant gene, indicating haploinsufficiency [18].

X-linked ID is one of the least recognized causes by physicians, emphasizing the importance of genetic investigation [2]. In our patient, initial suspicions were of congenital rubella and/or toxoplasmosis syndrome, as the results for these infectious diseases were positive in the child after birth and in the mother. The genetic investigation began with a karyotype, which revealed a pericentric inversion of chromosome 9, also found in the child’s father but without signs of malignancy. From there, the suspicion of 9q13 microdeletion syndrome arose, and a CMA test was requested, which did not report any alterations. Our last available resource was WES, which was requested and identified the disease-causing variant.

## Conclusion

We present an ultrarare case of MRXS99F with unique phenotypes and highlight the diagnostic challenges of this type of X-linked intellectual disability due to its resemblance to many other conditions. We emphasize the importance of genetic professionals, as well as both medical and multidisciplinary staff, in the investigation and diagnosis of rare and complex diseases. The incomplete penetrance of variants in the *USP9X* gene requires further investigation to be understood. This piece of information is crucial to provide accurate risk estimation and adequate genetic counseling for the families harboring LOF variants in *USP9X*.

## Data Availability

Data are unavailable due to privacy or ethical restrictions.

## References

[1] American Psychiatric Association. Diagnostic and Statistical Manual of Mental Disorders. Diagnostic and Statistical Manual of Mental Disorders. 5th ed. 2013;5(5).

[2] Meira JGC, Magalhães BS, Ferreira IBB, Tavares DF, Kobayashi GS, Leão EKEA. Novel USP9X variant associated with syndromic intellectual disability in a female: a case study and review. Am J Med Genet A. 2021;185(5):1569–74. 33638286 10.1002/ajmg.a.62141

[3] Olusanya BO, Gladstone M, Wright SM, Hadders-Algra M, Boo NY, Nair MKC, *et al*. Cerebral palsy and developmental intellectual disability in children younger than 5 years: findings from the GBD-WHO rehabilitation database 2019. Front Public Health. 2022;25:10. 10.3389/fpubh.2022.894546 PMC9452822 36091559

[4] Tarpey PS, Smith R, Pleasance E, Whibley A, Edkins S, Hardy C, *et al*. 2009. A systematic, large-scale resequencing screen of X-chromosome coding exons in mental retardation. 41(5):535–43. 10.1038/ng.367 PMC2872007 19377476

[5] Whibley AC, Plagnol V, Tarpey PS, Abidi F, Fullston T, Choma MK, *et al*. Fine-scale survey of X chromosome copy number variants and indels underlying intellectual disability. Am J Hum Genet. 2010;87(2):173–88. 20655035 10.1016/j.ajhg.2010.06.017 PMC2917707

[6] De Luca C, Race V, Keldermans L, Bauters M, Van Esch H. Challenges in molecular diagnosis of X-linked intellectual disability. Br Med Bull. 2020;133(1):36–48. 32043524 10.1093/bmb/ldz039

[7] OMIM - #300968 - Intellectual developmental disorder, X-linked 99, syndromic, female-restricted; MRXS99F—OMIM. Omim.org. 2016. https://www.omim.org/entry/300968?search=%23300968&highlight=300968. Accessed 28 Nov 2024.

[8] Orphanet: X-linked female restricted facial dysmorphism-short stature-choanal atresia-intellectual disability. Orpha.net. 2020. https://www.orpha.net/en/disease/detail/480880. Accessed 28 Nov 2024.

[9] Lenberg JL, Pretorius DH, Rupe ES, Jones MC, Ramos GA, Andreasen TS. Whole-exome sequencing reveals novel USP9X variant in female fetus with isolated agenesis of the corpus callosum. Clin Case Rep. 2019;7(4):656–60. 30997057 10.1002/ccr3.2051 PMC6452501

[10] De Laurentiis A, Ciaccio C, Erbetta A, Pinelli M, Nigro V, Pantaleoni C, *et al*. Periventricular heterotopia in a male child with USP9X missense variant. Am J Med Genet Part A. 2023;191(5):1350–4. 36680497 10.1002/ajmg.a.63123

[11] ClinVar. USP9X[gene]—ClinVar—NCBI. Nih.gov. 2024. https://www.ncbi.nlm.nih.gov/clinvar/?term=USP9X%5Bgene%5D&redir=gene. Accessed 28 Nov 2024.

[12] USP9X - DECIPHER v11.28. Deciphergenomics.org. 2024. https://www.deciphergenomics.org/gene/USP9X/patient-overlap/snvs. Accessed 4 Dec 2024.

[13] Nagata N, Kurosaka H, Higashi K, Yamaguchi M, Yamamoto S, Inubushi T, *et al*. Characteristic craniofacial defects associated with a novel USP9X truncation mutation. Human Genome Var. 2024;11(1):21. 10.1038/s41439-024-00277-w PMC11099082 38755172

[14] Li D, March ME, Wang T, Merengwa V, Finoti LS, Schrier SA, *et al*. Exome and RNA-Seq analyses of an incomplete penetrance variant in USP9X in female-specific syndromic intellectual disability. Am J Med Genet A. 2022;188(6):1808. 35253988 10.1002/ajmg.a.62715

[15] Gargano M, Matentzoglu N, Coleman B, Addo-Lartey EB, Anagnostopoulos AV, Anderton J, *et al*. The human phenotype ontology in 2024: phenotypes around the world. Nucleic Acids Res. 2023;52(D1):D1333–46. 10.1093/nar/gkad1005 PMC10767975 37953324

[16] Reijnders MRF, Zachariadis V, Latour B, Jolly L, Mancini GM, Pfundt R, *et al*. *De novo* loss-of-function mutations in USP9X cause a female-specific recognizable syndrome with developmental delay and congenital malformations. Am J Human Genet. 2016;98(2):373–81. 26833328 10.1016/j.ajhg.2015.12.015 PMC4746365

[17] Vianna EQ, Piergiorge RM, Gonçalves AP, Santos JM, Calassara V, Rosenberg C, *et al*. Understanding the landscape of X-linked variants causing intellectual disability in females through extreme X chromosome inactivation skewing. Mol Neurobiol. 2020;57(9):3671–84. 32564284 10.1007/s12035-020-01981-8

[18] Agazzi C, Magliozzi M, Iacoviello O, Palladino S, Delvecchio M, Masciopinto M, *et al*. Novel variant in the USP9X gene is associated with congenital heart disease in a male patient: a case report and literature review. Mol Syndromol. 2022. 10.1159/000527424. 10.1159/000527424 PMC10090979 37064340

